# Postnatal Innate Immune Development: From Birth to Adulthood

**DOI:** 10.3389/fimmu.2017.00957

**Published:** 2017-08-11

**Authors:** Anastasia Georgountzou, Nikolaos G. Papadopoulos

**Affiliations:** ^1^Allergy and Clinical Immunology Unit, 2nd Pediatric Clinic, National and Kapodistrian University of Athens, Athens, Greece; ^2^Division of Infection, Inflammation and Respiratory Medicine, The University of Manchester, Manchester, United Kingdom

**Keywords:** innate immunity, postnatal development, innate ontogeny, immune trajectories, immune-related diseases

## Abstract

It is well established that adaptive immune responses are deficient in early life, contributing to increased mortality and morbidity. The developmental trajectories of different components of innate immunity are only recently being explored. Individual molecules, cells, or pathways of innate recognition and signaling, within different compartments/anatomical sites, demonstrate variable maturation patterns. Despite some discrepancies among published data, valuable information is emerging, showing that the developmental pattern of cytokine responses during early life is age and toll-like receptor specific, and may be modified by genetic and environmental factors. Interestingly, specific environmental exposures have been linked both to innate function modifications and the occurrence of chronic inflammatory disorders, such as respiratory allergies. As these conditions are on the rise, our knowledge on innate immune development and its modulating factors needs to be expanded. Improved understanding of the sequence of events associated with disease onset and persistence will lead toward meaningful interventions. This review describes the state-of-the-art on normal postnatal innate immune ontogeny and highlights research areas that are currently explored or should be further addressed.

## Introduction

Neonatal immunity is often characterized as “immature,” mostly due to impaired adaptive responses in comparison to adults, especially concerning the deficiencies in B-cell function and antibody production, but also the limited in magnitude T-cell responses and their partial bias to Th2-type (1). Nonetheless, functional deficiencies also extend to the innate branch of the immune system and several differences between neonates and adults concerning barriers, cell populations, and the complement have been identified (2–4). In the last few years, with the discovery of toll-like receptors (TLRs) and other pattern-recognition receptors (PRRs) and our improved understanding of innate immune recognition and function, it has become obvious that the innate immune system of neonates differs in many aspects from that of adults (3, 5). The fact that significant morbidity and mortality from infection extends beyond the neonatal period reflects the persistence of immune immaturity in several defense mechanisms at least throughout infancy and early childhood and has led to remarkable research on immune development, in order to improve anti-infective strategies and to design novel vaccines (6, 7). In specific, innate immune ontogeny is a field of active research, as it has been recognized that innate immune activation, apart from being essential for protection against infectious agents in the beginning of life, may regulate and shape adaptive responses (8, 9) and may be implicated in the pathogenesis of inflammatory, autoimmune, and allergic diseases (10, 11). Therefore, several investigators have attempted to gain insight into innate immune ontogeny, mainly by defining age-dependent changes in PRR-mediated innate immune responses and to estimate the impact of genetic factors and environmental exposures in this developmental process.

The innate immune system comprises of mucosal and epithelial barriers, cells, such as neutrophils, antigen-presenting cells (APCs), mast cells, eosinophils, and natural killer (NK) cells, and soluble factors (SF) including antimicrobial proteins/peptides, cytokines, chemokines, acute phase proteins, and the complement (12). Specific developmental changes concerning components of the innate immune system, such as cell populations, barriers and the complement have been described several years ago (13–16). More recently, new sophisticated techniques such as multiplexing technology, have permitted detection in small specimen volumes of a broad range of molecules, mainly cytokines and description of age-dependent changes in the response pattern to multiple pathogen-associated molecular patterns (17). As knowledge in this field is expanding, this review will provide an overview and synopsis of the state-of-the-art on the postnatal ontogeny of the human innate immune system, describing developmental changes in barriers, cells and SF.

## Postnatal Development of Innate Immune Components

### Barriers

The skin and the mucosal immune system are the first line of defense against pathogens.

#### Skin

The skin forms a barrier against physical, biological, and chemical stress and is a fundamental component of innate immunity. The epidermis and in particular its top layer, stratum corneum (SC), mediates multiple protective functions dependent on its structural, biochemical, and cellular mechanisms. Distinct components of the defensive barrier include the epidermal thickness and integrity, the acidic pH, the skin microbiome, several antimicrobial peptides (AMPs), and the resident immune system cells (18, 19).

Recent research has yielded evidence that, although the skin of full-term neonates is anatomically mature with all layers present, significant age-related structural and functional alterations occur at least throughout the first years of life (14, 20). First, epidermal thickness seems to increase progressively from birth to adulthood. Children <2 years have smaller corneocytes and keratinocytes than adults (21). Both SC and the supra-papillary epidermis were found significantly thinner (on average 30 and 20%, respectively) in children aged 6–24 months in comparison to adults, when calculated by confocal laser scanning microscopy (21) and a linear increase in skin thickness with age in subjects 1–20 years was observed using pulsed ultrasound (22). Males eventually develop thicker epidermal and dermal layers (23). Collagen fiber density in the upper part of dermis has also been reported to increase up to the age of 30–40 years and then starts to decrease, whereas elastic fiber density increases during the first decade, then transiently drops, before reaching peak levels in adulthood (24). Nonetheless, some investigators found no significant differences in SC thickness between infants and young adults, with measurements performed in *ex vivo* histological samples (25). The water content of the skin is of essential significance to the barrier function as it regulates, among others, the activity of hydrolytic enzymes that are involved in SC maturation and corneocyte desquamation. Neonatal skin is dry and relatively rough, but smoothens during the first month of life, as the SC hydration progressively increases (26, 27) and even exceeds the hydration level found in adults at some point during the first 2 years of life (28–30). Transepidermal water loss (TEWL) describes the amount of water loss through the epidermis with evaporation and is one of the methods used to assess the quality of the skin’s barrier function. There are discrepancies among studies that compare TEWL rates in infants, children, and adults (14, 20). Values comparable to adult levels were found in several anatomical sites of infants (such as the chest, back, and abdomen), whereas pronounced differences were observed when testing others (such as volar and extensor forearm, flexor upper arm, lateral upper leg, and buttock) (31). This could be due to multiple factors that are known to affect those rates, such as local hemodynamics, degree of corneocyte formation, and SC lipid content (25); nonetheless, the large intersubject variability particularly observed among young infants may be indicative of an immature barrier developing with variable rates at different sites during infancy (14, 29). In the same direction, studies indicate that the water-handling properties of the skin, measured by tools other than TEWL, have also not reached adult levels up to the first year of life (29).

The acidic character of the skin surface pH, ranging from 4.5 to 6.7, has long been appreciated as a significant defensive mechanism, therefore named the “acid mantle” of the skin (14). Newborns have a skin pH closer to neutral, ranging from 6.11 to 7.5, depending on the site of measurement (14, 31). The skin acidity decreases significantly during the neonatal period with major alterations occurring in the first 1–4 postnatal days. Subsequently, pH continues to drop during the first 3 months, remaining higher compared to adults. In contrast to adult skin, pH levels in infancy differ substantially between studies, according to age and anatomic region. Consequently, the age at which pH levels are stabilized and become similar to adult levels is not unanimously defined (14, 26, 27, 31).

The skin, apart from being a physical barrier, is also an immunological barrier, as it is rich in T lymphocytes and innate immune cells, such as macrophages, neutrophils, innate lymphoid cells, and dendritic cells (DCs), including dermal DCs and the epidermal Langerhans cells. Keratinocytes are also important players in innate protection as they express a broad range of PRRs (18, 19, 32, 33). Data on age-related changes of innate cells’ population in the human skin are few. Dermal DCs, Langerhans cells, and macrophages were shown by flow cytometry and immunofluorescence to have populated the skin before mid-gestation and to progressively present a high degree of maturity at a prenatal stage, although fewer in numbers in comparison to adults (34, 35). The same has been shown for Langerhans cells by immunolabeling and histochemical techniques (36). Postnatal data are lacking; however, there is evidence of differential expression and function of some TLRs in the developing skin. Iram et al. showed using quantitative real-time PCR that prenatal skin expresses the same spectrum of TLRs as adult skin; nonetheless TLR1 and 3 expression was significantly higher in infants (aged 3–10 months) and children (5–12 years) than in adults (18–31 years) and an opposite trend was observed for TLR6 expression (37). The same group also found that neonatal keratinocytes secreted higher levels of the chemokines CXCL8 and CXCL10 and of tumor necrosis factor α (TNF-α) upon TLR3 ligation with poly(I:C) compared to adult keratinocytes, but did not provide information for intermediate time points. Recently, Kinn et al. observed progressively decreasing skin concentrations of interferon (IFN)-α2 using multiplexed fluorescent bead-based immunoassays between groups of young (24.3 ± 2.8 years), middle-aged (56.6 ± 4.6 years), and elderly (72.9 ± 3.0 years) adults, but no data on skin IFN production in younger subjects exist (38).

Antimicrobial peptides are important elements of the innate immune system. In the human skin, AMPs are produced by a broad range of cells, including keratinocytes, sebocytes, and mast cells, but also locally recruited neutrophils and NK cells. Among skin AMPs, cathelicidins, and β-defensins are the most well characterized, and it is known that they are secreted at low levels under normal conditions in adults (39). Dorschner et al. found that cathelicidin (LL-37) skin expression was significantly increased in the perinatal period (40) compared to adult life and the same was observed for the antimicrobial proteins lysozyme and lactoferrin (41). Probably this increased expression during neonatal period represents a compensatory innate defense mechanism until the maturation of cellular immune responses, but the exact point at which the production of antimicrobial substances in the skin is reduced to adult levels remains unaddressed.

A diverse, site-specific, microbiome exists on human skin and it has recently been shown that commensal bacteria may modulate host immune responses and induce protection against pathogens (42–44). Furthermore, microbial communities may exhibit temporal variations, in response to environmental stimuli (45). Little is known about the skin microbiome development. It was shown, though, that vaginally delivered neonates acquired bacterial communities resembling their mother’s vaginal microbiota, whereas neonates born with caesarian section harbored bacterial communities similar to those found on the skin surface (46). In newborns, bacterial communities appear to be undifferentiated across various body sites (46), but within the first trimester a site-specific evolution begins (47). Moreover, during the first year of life, the composition of skin microflora changes, presenting an increase in diversity with age (47). Shifts in the skin microflora occur later in life, as well, and interestingly, significant changes have been associated with progressive sexual maturation (48).

#### Oral and Gastrointestinal Mucosae

The formation of the mucosal epithelial barrier is initiated during the 10th gestational week and anatomically the structures of the mucosal immune system are developed by the 28th gestational week. However, unless an intrauterine infection has occurred, the development of effective immunity takes place in the postnatal period. Two major factors are believed to influence the postnatal immune maturation of the gastrointestinal tract: bacterial colonization and oral feeding (49, 50).

A first significant maturational step is the closure of the mucosal epithelial membranes. The increased intestinal permeability attributed to the immaturity of intercellular tight junctions during the fetal period is beneficial, as it facilitates the exchange of bioactive molecules between amniotic fluid and the fetus. This membrane deficiency is still present at birth and the immediate postnatal period (51, 52). Nevertheless, a rapid closure of mucosal epithelial membranes is observed in the gut within the first postnatal week, as indicated by the drop in lactulose/mannitol intestinal permeability test (53), and this procedure is promoted by the ingestion of colostrum and orchestrated by several regulatory factors. Interestingly, it has been observed that colostrum deprivation results in the delayed closure of mucosal membranes (53, 54), and this prolonged period of increased mucosal permeability could be linked to higher occurrence of infections and atopic diseases in non-breastfed children (49). Membrane closure also occurs in the upper gastrointestinal tract, as indicated by the gradual loss of IgG from saliva or nasopharyngeal secretions (55, 56); this procedure appears to progress over the first 2 months of life. The closure of membranes is followed by progressive luminal epithelial growth, with villous and crypt hyperplasia, which is believed to be accelerated throughout infancy and especially during the weaning period (57). Nonetheless, the mucosal barrier is still considered rather inadequate up to the second year of life, probably because of the concomitant immaturity of the secretory immunity components (58).

Apart from the epithelial barrier, significant innate protection is conferred by mucin glycoproteins, lining the epithelium and serving as a trap for microbes and potential immunogenic particles, as well as by AMPs, such as defensins, cathelicidin (LL-37), lysozyme, lectins and, finally, by other proteins like lactoferrin and amylase, that altogether alter antigen exposure by killing or inactivating microbes and modifying potentially immunogenic substances (59–62). Scarce human data suggest that the mucin profile at birth is similar to the adult (63), although in other mammals the production of intestinal mucins undergoes developmental changes, correlating with successional changes in the indigenous microflora and multiple other factors, such as microbes, microbial products, toxins, cytokines, hormones/neuropeptides, and growth factors (64, 65). Little is known about the age-related changes in the concentrations of the aforementioned antimicrobial substances in the human gastrointestinal tract, but for most of them a postnatal rise has been observed. Since the early 90s, it has been reported that the levels of amylase, lysozyme, and lactoferrin in salivary glands of fetuses after the sixth gestational month are comparable to those of adults. Nevertheless, a temporary decrease in the level of all three proteins is observed after birth, followed by progressive augmentation to peak levels during the second to fourth month and, finally, stabilization to adult levels around the sixth month of age (49, 66). Ben-Aryeh et al. found a positive age correlation of salivary amylase activity in a population with age range from 7 months to 63 years, with the most significant rise occurring between the infant and the toddler group (67). In the lower gastrointestinal tract, lysozyme production was also reported to increase postnatally, as significant higher levels were found in feces of 7-day-old neonates than in meconium (68) and recently, the same was reported for fecal lactoferrin levels, that increased from birth to 1 month (69).

The rise in AMP secretion during the neonatal period has been attributed to the augmentation in the number of AMP-producing cells and to gene induction by food components or the gut microbiome. As the intestinal epithelial growth is intense during infancy and the transition to an adult-like bacterial microflora is incomplete before the third year (70), one can assume that there are significant variations in AMP production at least during the first 2 years of life (71). Indeed, there are reports of developmentally regulated expression of AMPs in mice, like ANG4 and REG3γ, that reach adult levels during weaning or CRAMP, the murine intestinal homolog of human cathelicidin LL-37, that is highly expressed in neonatal epithelium and becomes less abundant during the postnatal period, but these data have not yet been confirmed in humans (60, 72). It has been shown though, that feces of neonates contain higher levels of LL-37 than meconium (68) and Davidopoulou et al. observed a positive correlation with age of the salivary LL-37 concentration in children aged 2–18 years (73). Developmental expression has also been confirmed for human defensin family members, HD-5 and HD-6, as their mRNA levels are lower in fetal life compared with the term newborns and adults (74) and, in particular, some have reported that enteric defensin mRNA levels in the second trimester of gestation are 40- to 250-fold less than those observed in the adult gut (75). Finally, Malcolm et al. found that the salivary concentrations of calprotectin and human neutrophil peptides 1–3, also members of the AMP family, increased with age in children aged 1–3 years, in parallel with increasing bacterial colonization (76). On the contrary, the levels of stool calprotectin were reported at birth to be comparable to those of patients with inflammatory bowel disease (77), then, according to novel findings, they exhibit a downward trend and finally stabilize to adult levels after the fourth year of life (78–80).

Gastric pH, determined by the basal acid output of the stomach, could also be considered as a contributor in innate defenses, as it facilitates protein digestion. In neonates the acid output is low and an age-related augmentation is observed, with adult levels reached only after the second year of life (81).

Very little is known about the postnatal development of innate cells at mucosal sites in humans. It is likely that the endothelial adhesion molecules required to direct phagocytes to the intestine are already present before birth (49). It seems that the intestinal innate cellular composition displays a rather mature phenotype at birth, with the exception of DCs that appear to expand postnatally, along with the expansion of the intestinal lymphoid tissues (49, 82–84), although there have been reports of their presence in the lamina propria of fetal intestinal samples (85).

Moreover, there is a paucity of data regarding the postnatal alterations of epithelial innate immune signaling in humans. It is known that the intestinal epithelial cells express a wide array of receptors such as TLRs, but the relevant literature has mainly focused on early adaptive regulations that prevent excessive inflammation due to microbial exposure during the immediate postnatal period. These mechanisms include a reduction in TLR surface expression, as it has been shown for TLRs 2 and 4 (86, 87), developmental changes in post-receptor signal transduction complexes that are linked to the activation of the NFκB pathway, such as the underexpression of MyD88 and TRAF6 (87) and the increase in IκB expression (88), and consequently the reduced transcription of inflammatory cytokines, such as interleukin (IL-) 8 (86, 87, 89). Furthermore, an enhanced expression of surface and intracellular negative regulatory molecules implicated in TLR signaling, such as A20, single immunoglobulin IL-1 receptor-related molecule (SIGIRR), interleukin 1 receptor associated kinase-M, and toll-interacting protein (TOL-LIP) has been described in mature human neonatal epithelial cells (86, 87). Beyond the neonatal period, Pott et al. reported age-related differences in TLR3 expression of the intestinal epithelium, with a significant upregulation noted in children of 5 years and older, correlating with the enhanced resistance to rotavirus infection in this age group (90). Postnatal epithelial responses have been described in greater detail in mice, with significant changes occurring during weaning period (91); nonetheless, this subject remains largely unaddressed in humans.

#### Respiratory Mucosa

The upper and lower airways are the largest epithelial surfaces of our body exposed to the outer environment. Major innate protective mechanisms of the respiratory system include the cough reflex, the mucociliary apparatus, and several secreted antimicrobial substances, such as lysozyme, lactoferrin, defensins, and surfactant proteins. Epithelial cells provide a mucosal barrier and are involved in multiple defense mechanisms by recognizing microbes through PRRs and by interacting with innate cells, notably alveolar macrophages (AMs), DCs, and neutrophils. A multitude of cytokines and chemokines are released locally upon stimulation of the respiratory mucosa and early immune interactions are thought to be of major importance to the development of diseases, such as asthma (92, 93). Nonetheless, little is known about the ontogeny of innate immune mechanisms in the human lung.

The mucociliary clearance has been evaluated by calculating nasal mucociliary clearance time in the saccharin test and was found impaired in children by several groups (94, 95), with the impairment being more pronounced in preschool years (94). On the contrary, investigators using alternative techniques to assess mucociliary clearance, such as radioisotopy (96) or the measurement of ciliary beat frequency in biopsies after ciliogenesis in culture by photometry (97), found no correlation with age.

The presence of defensins and cathelicidin LL-37 in the human lung has been documented by several groups (98, 99). Human defensin b-2 production has been assessed in tracheal aspirates from newborns and was found more abundant with advancing gestational age (100), but no data on postnatal developmental expression of this or other AMP in the human airways exist.

Our knowledge on the immune cellular profile of the lungs in the beginning of life is still expanding. Relevant investigations could largely contribute to our understanding of the responses to infectious or environmental agents, but are limited by the difficulty in obtaining lung tissue in healthy children. It appears that the lungs are devoid of immunocompetent or inflammatory cells in the intrauterine stage (15). Innate immune cells seed the airways during early life and there is good evidence that the profile of lung leukocytes does not change significantly after 3 years of age. Epithelial HLA-DR expression has been reported absent in fetal tracheal epithelium, but was documented during the first week after delivery and found to increase from the first to the second week of life. These data suggest a postnatal seeding of the airways by cells, such as macrophages (101). The total leukocyte and differential counts in the bronchoalveolar (BAL) fluid of children did not vary between two age groups of normal children, 3–8 and 8–14 years, and were similar to values reported in adults (102). Ratjen et al. also did not find significant difference in the count of BAL fluid macrophages and lymphocytes in children aged 3–16 years, using linear regression analysis (103). In line with the above, Heier et al. reported that the density and location of APCs, which were mainly CD68+ macrophages and CD11c+ myeloid (m) DCs, in the airway mucosa of children older than 2 years, appears to be similar to those observed in adults (104). Interestingly, increased numbers and percentages of macrophages have been reported in the BAL fluid of healthy young children <2 years old, in comparison to older children (105), whereas others found a higher percentage of total leukocytes and neutrophils in healthy subjects <3 years, than in older children and adults (106). The presence and development of mature mucosal DCs during early childhood have been investigated by several groups. Earlier reports based on HLA-DR labeling and morphology examination of autopsy material (paraffin embedded tissues) from infants that had died from sudden infant death syndrome, trauma or respiratory tract infections and older subjects (children and adults), suggested that DCs are not constitutively present in the human tracheobronchial mucosa in the first year of life, and that their influx occurs progressively during infancy, triggered by infectious stimuli (107). However, more recent reports from subjects with (108) or without (109) respiratory disease indicate that DCs are present in the tracheal mucosa during infancy, though in significantly lower densities compared to samples from older subjects. Of note, in one of the studies, the total number of HLA-DR+ cells, among which approximately 50% were CD68+ macrophages and the remainder various subsets of DCs, increased significantly with age, between 4 and 23 months (108).

Apart from changes in numbers, there seem to be functional differences regarding lung innate cells in early life. Significant defects in the AM function during early childhood have been identified by Grigg et al.; before 2 years of age they express less HLA-DR, produce less IL-1 and TNF-α upon TLR4 stimulation and are impaired in their ability to reduce nitro blue tetrazolium (105). Age-related enhancement of the functional capacity of respiratory tract DCs has been demonstrated in animals (110), but no such data exist in humans.

### Cells

Circulating cells of the innate immune system include both myeloid cells, such as monocytes, DCs, granulocytes (neutrophils, eosinophils, basophils), and innate lymphoid cells, including NK cells (111). All innate blood cells are present in early life, but quantitative and qualitative differences have been observed between neonates and adults (3, 4, 17, 112–117).

It is well established that the normal range of total white blood cell (WBC) and differential counts varies with age. Healthy full-term neonates have a high WBC count with relative neutrophilia, and by 2 weeks of age there is a significant decline in leukocyte counts, followed by gradual decrease throughout childhood years to reach adult levels at around the 21st year. Furthermore, by the second postnatal week through preadolescence, lymphocyte predominance is observed, whereas during teenage years, neutrophils become the predominant WBC in relative percentage, as in adults (13, 118, 119).

#### Granulocytes

Quantitative differences of neutrophils (polymorphonuclear leukocytes, PMN), the most frequent granulocytes, across the lifespan have been sufficiently studied. The neutrophil count peaks 12 h post-partum, then decreases and stabilizes from the fifth postnatal day throughout the neonatal period (120, 121). During childhood, as lymphocytes predominate in percentage, neutrophil numbers relatively decrease, and then rise again in adolescence (122), but variations in mean numbers among age groups are less significant than those observed in the first postnatal days. Neutrophils, although more abundant in newborns, present significant functional defects in early life (123). It has long been known that neonatal PMN exhibit reduced adherence, chemotaxis, and migration, probably linked to reduced expression of cell membrane adhesion molecules (i.e., b2 integrins, such as Mac-1 and LFA-1, and selectins) (124–127). There is great divergence in studies concerning the postnatal maturation of chemotaxis. Several researchers found that maturation to adult levels was achieved rapidly, by the second to fourth week of age (125, 128), while others found that chemotaxis was significantly defective at 6 months (129), before 12 months (130), and at 2–5 years (131), or still decreased in teenagers under 16 years when compared to adults (132, 133). Of note, Storm et al. studied the postnatal maturation of integrin Mac-1 expression of PMN in infants and found it 50% reduced at 1–2 months of age; thereafter, it steadily increased to reach adult levels by the end of first year (134). Moriguchi et al. found that the lower, compared to adults, L-selectin expression on human neonatal neutrophils normalizes within the first week of life (135), while Kim et al. observed that the decreased expression persisted for at least 4 weeks (136). Neonatal neutrophils also exhibit reduced microbicidal activity, especially under suboptimal conditions, such as decreased opsonin concentrations or high bacterial charge (3, 4, 17, 114, 137). In optimal circumstances of full opsonization, the ingestion of Gram-positive and Gram-negative bacteria has been found normal, whereas *Candida* ingestion was impaired, up to the second postnatal week (123). PMN phagocytosis assessed by a technique using the yeast *S. cerevisiae* reached normal adult levels around the first year of life (138).

Basophil counts have been reported to be elevated during early life (139), then, by one group to be stable from 6 months to 18 years (122), whereas others observed a decline with age from 4 to 19 years (140). The functional maturation of basophils has not been studied up to now.

Neonates also have relatively high eosinophil counts, but a decline is observed with age (113, 122, 140). An age-related change in circulating eosinophil degranulation in response to IL-5 stimulation has been observed between younger and older adults (significantly decreased in the older group), but the effector functions of eosinophils in the beginning of life have not been studied (141).

#### Antigen-Presenting Cells

Neonates have higher mean monocyte counts than adults. During the first month of life there is an augmentation in the average proportion of monocytes (up to 9% of total WBC); their count slightly declines subsequently and reaches adult levels during third to fifth month of age (122, 140) or, according to others, at some later time point during early childhood (142), with no significant changes occurring thereafter. Neonatal monocytes have been reported to display impaired chemotactic activity and altered phagocytosis (112, 137). At 6 months of age, monocyte chemotaxis was found decreased in comparison to adults (129) and two other groups observed that it was still significantly low through age 5 (132) or 6 years (131) and remained moderately reduced until age 10 (132). The difference between adults and children was more pronounced before the age of 12 months (131). The expression of the adhesion receptors LFA-1 and LFA-2 (CD2) has also been studied in human mononuclear cells (MCs) from cord blood, children, and adults and was found to be at adult levels in children older than 6 months (143). Data on phagocytic capacity maturation are scant and in a study assessing phagocytosis of the yeast *S. cerevisiae* by monocytes among different age groups there were no significant variations (138). Phenotypic maturation has been observed in monocytes as Nguyen et al. assessed their surface expression of CD80 and HLA-DR molecules, which are important to their immune interaction with T lymphocytes. CD80 expression on monocytes after TLR4 and 9 stimulation reached adult levels by third month of age, whereas HLA-DR expression was similar to that of adults after the sixth month of life (144).

Age-related differences have been also observed in DC counts and subset composition from infancy to adulthood. In a study by Orsini et al., the total DC number showed an inverse correlation with age across the lifespan, and so did the absolute numbers of circulating mDCs and plasmatocytoid DCs (pDCs) (145). Studies that have focused on changes occurring during childhood (up to 18 years of age) showed an age-dependent significant decline either for both DC subsets (146, 147) or for pDCs only (148–150). Nonetheless, a study with a small group of children did not reveal age-dependent correlations for either pDC or mDC subsets (151) and another found similar pDC content in adult and neonatal blood and increased mDC content in adult blood (5). It has been observed that at birth pDCs outnumber mDCs, so that the pDC-to-mDC ratio is inversed in comparison to adults (152, 153). Jyonouchi et al. reported that the pDC-to-mDC ratio stabilized to the usual adult level 1:2 around the 10th year of age (149). The use of different combinations of markers for the identification of DC subsets among the studies probably accounts for the discrepancies between published data.

Phenotypic age-related changes have been observed in mDCs and pDCs, in terms of HLA-DR and CD80 expression, upon TLR4 and 9 stimulation. For mDCs, adult-level HLA-DR and CD80 expression was already reached in 3-month-old infants, whereas for pDCs in 6- to 9-month-aged subjects (144). During the last few years, it has become obvious that major functional maturation of circulating monocytes and DCs occurs during the first years of life, reflected by changes in the responsiveness to TLR stimulation (154), although the pattern of TLR expression by APCs has been reported to be similar between neonates and adults (116, 155) and among children of different ages (156). These changes, in terms of measurement of cytokine production, represent at least to a certain extent how the innate immune response to microbial exposure evolves and will be described in detail below.

#### NK Cells

The highest counts of NK cells are found in cord blood but they decline significantly (by 2–3 times) during the first postnatal days. Their levels further decrease progressively throughout infancy and early childhood, to reach adult levels around the fifth year of age (157). There are no significant changes of the NK cell circulating subsets, CD56 bright, and CD56 dim, until adulthood, apart from a slightly higher proportion of CD56 bright NK cell numbers in neonates. Of note, CD56 bright cells are poorly cytotoxic in comparison to CD56 dim cells, but efficiently secrete cytokines such as IFN-γ and TNF-α (157, 158). Regarding functional maturation, one group assessed the cytotoxicity of NK cells against K562 cells throughout childhood and found it extremely low during neonatal period, but increased rapidly between first and fifth month of life to almost adult levels (159). More recently, it was shown that the responses of NK cells are determined by the balance between activating and inhibitory signals from relative cell surface receptors and several phenotypic changes on NK cells regarding the expression of these receptors have been reported during the first years of life (158). A progressive decrease in the expression of the inhibitory C-type lectin receptor NKG2A has been reported from newborns to the second decade of life (158, 160), with a parallel upregulation of killer cell immunoglobulin-like receptor (KIR) expression during the first decade of life and especially in the first 2 years (158). The neonatal KIR− NKG2A+ NK phenotype is associated with a cytokine-producing NK function rather than cytotoxicity, whereas the KIR+ NKG2A− NK cell expresses markers associated with maturity. The aforementioned changes during the first two decades of life correlate with terminal differentiation of NK cells, as defined by reduction in proliferation capacity, response to cytokines, and expression of activation markers (161). Another group found that cord blood NK cells express in lower proportion the inhibitory leukocyte immunoglobulin-like receptor-1 and its expression rises to adult levels around the age of five (162). On the contrary, a higher expression of the activating receptors NKG2D, as well as NKp30 and NKp46 (belonging to the family of natural cytotoxicity receptors) was observed in the beginning of life in comparison to later time points in infancy and early childhood and it has been hypothesized to enable NK implication in defense responses during a time period when innate immunity is of critical importance for survival (162).

### Soluble Factors

#### Complement

The complement system is among the most extensively studied non-cellular elements of the innate immune system. It has long been observed that the neonatal serum contains lower levels of complement proteins, notably 10–80% of adult levels, depending on the protein and on the methodology applied, so that both the classic and alternative pathways of the complement cascade are affected (163–165). C8 and especially C9 are the most markedly reduced at birth (166, 167). Moreover, functional assays have shown that the complement system exhibits at birth lower opsonizing capacity, relatively impaired chemotaxis and reduced lytic function (117, 166). Age-related changes in serum concentrations of C3 and C4 have been studied by many groups and seem to reach adult levels within 3–6 months (165, 168, 169). Similar results have been reported for proteins C2, C6, and factor B (169), while for C5 some observed the same pattern (168, 169) and others found a progressive reduction with age (170). The levels of mannan-binding lectin have been reported to rapidly increase during the first days (171, 172) or months (173) of life, but the age at which they stabilize varies significantly among studies, from 3 months (173) to adolescence (172). C1q and properidin levels seem to reach adult levels after the first year of life, around 18–21 months of age (169, 174). Serum concentration of CH50 has been reported to adult levels at 6 months (168), although in studies engaging a broader age spectrum, from birth to adolescence (170) or analyzing older children, from 3 to 14 years, there were no significant age-dependent variations (175). The ontogeny of some regulatory proteins of the complement system, such as factor H (fH), factor I (fI), C4b-binding protein (C4 BP), and vitronectin (also known as protein S), has also been assessed (174). De Paula et al. found that fH and fI levels reached adult levels in the first year of life. For C4 BP, there was a significant rise among 6- to 13-year-old children, whereas adults presented significantly higher vitronectin levels than children of all ages.

The detection of complement components in serum may not be reflecting accurately its defense capabilities. Nonetheless, the observed age-related differences offer an insight of the maturational process, since the synthesis of complement proteins appears to develop simultaneously and in sequence with total hemolytic activity (168). Moreover, the measurement of CH50 has been considered a satisfactory screening method to assess the integrity and function of the entire complement (170).

#### Cytokines and Immune Pathways

Differences between neonatal and adult monocyte and DC responses to stimulation *via* TLRs, as well as the underlying molecular mechanisms have been reviewed elsewhere (3, 114, 115, 154). Most studies suggest that basic features of the neonatal innate response are the impaired production of antiviral type 1 IFNs and Th1-related cytokines, such as TNF-α, IFN-γ and IL-12p70, with the concomitant increased production of anti-inflammatory IL-10 and Th2/Th17 supporting/promoting cytokines IL-6 and IL-23. Moreover, at the single cell level, neonatal monocytes and DCs are less polyfunctional, that is to say they exhibit reduced capability of simultaneous production of multiple cytokines upon immune stimuli (5) and this deficiency seems to persist throughout infancy (176). Nevertheless, neonatal TLR responses are not globally impaired, as certain stimuli, such as TLR8 ligation, may elicit robust, adult-like responses from monocytes and DCs (177, 178).

Recently, several longitudinal or cross-sectional studies have sought to shed light on the postnatal ontogeny of TLR-mediated cytokine responses, using either whole blood assays or MC cultures. The most important characteristics of these studies are illustrated in Table 1. Although there are important variations among published data, partially attributed to differing study design and laboratory methods, important information has emerged, showing that the developmental pattern of cytokine responses is non-linear but age- and TLR specific. Furthermore, Smolen et al. in a comparative study showed substantial differences in cytokine production among 2-year-old children from different continents, with more pronounced variations between the responses of South African subjects vs those from Ecuador, Belgium, and Canada (179). Significant differences in cytokine (TNF-α and IL-10) production upon TLR ligation have also been observed between Dutch and Gabonese school-aged children (180). Moreover, Tulic et al. observed significant differences in the developmental trajectories of innate immune function between children with allergic disease and their non-allergic peers (181). Those distinct responses suggest the influence of genetic factors and environmental exposures on immune status and are indicative of differential developmental innate immune trajectories among different populations or different subgroups of the same population. Despite the discrepancies in literature, several points of consensus regarding the developmental pattern of cytokine production after TLR stimulation have emerged. A number of studies from western countries suggest the postnatal increase of Th1 supporting cytokines and type I IFN during infancy, with a parallel decrease of Th17 and anti-inflammatory cytokines (144, 176), whereas opposite developmental trends have been observed in an African population (182).

**Table 1:** Studies assessing the postnatal ontogeny of toll-like receptor (TLR)-mediated cytokine responses.

Culture system	Pattern-recognition receptor pathways	Outcome	Age range	Location of study	Type of study	Reference
PBMCs (cryopreserved)	TLR4	IL-10, IL-12	0–12 years, adults	Australia	Cross-sectional	Upham et al. (187)
Whole blood	TLR4	IL-8, IL-12	0–19 years, adults 20–40 years	Japan	Cross-sectional Allergic vs non-allergic	Itazawa et al. (196)
Whole blood	TLR4	IL-12	1–96 months, adults	Germany	Cross-sectional	Härtel et al. (186)
PBMCs (cryopreserved)	TLR4	IL-6, IL-10, IL-12, IL-18, TNF-α, IL-23, myxovirus resistance protein A [induced by type I interferon (IFN)], IFN-γ	0–13 years, adults 23–57 years	Australia	Cross-sectional	Yerkovich et al. (188)
Whole blood	TLR3, TLR4, TLR7, TLR9	IL-10, IL-12p70, IFN-α	0–1 month, adults	Netherlands	Cross-sectional	Belderbos et al. (184)
Whole blood	TLR4	IL-5, IL-8, IL-10, TNF-α, IFN-γ	0–1 year, adult mothers	Finland	Longitudinal and adult mothers (atopic and non-atopic)	Lappalainen et al. (190)
PBMCs (fresh)	TLR4, TLR9	IL-10, IFN-α	0–18 months, adults	Belgium	Longitudinal (partially) and adults	Vosters et al. (183)
Whole blood	TLR4, TLR9	IL-1β, IL-6, IL-8, IL-10, IL-12p70, TNF-α, IP-10, CXCL9 (MIG), IFN-γ	0–12 months, adults	Belgium	Longitudinal (partially) and adults	Nguyen et al. (144)
PBMCs (fresh)	TLR2/1, TLR3, TLR4, TLR7/8, TLR9	IL-1β, IL-6, IL-10, IL-12p40 and p70, TNF-α, IL-23, IFN-α, IFN-γ	0–2 years, adults 23–48 years	North America	Longitudinal and adults	Corbett et al. (176)
PBMCs (cryopreserved)	TLR2, TLR3, TLR4, TLR5, TLR2/6, TLR7/8, TLR9	IL-1β, IL-6, IL-10, IL-12, IL-13, TNF-α, IFN-γ	0–5 years	Australia	Longitudinal Allergic vs non-allergic	Tulic et al. (181)
Whole blood	TLR1/2, TLR3, TLR4, TLR5, TLR6/2, TLR7, TLR8, TLR9	IL-5, IL-6, IL-8, IL-10, IL-13, TNF-α, IFN-γ	6–60 months	Ecuador	Cross-sectional Urban vs rural environment	Teran et al. (193)
Whole blood	TLR1/2, TLR3, TLR4, TLR5, TLR2/6, TLR7, TLR8, TLR9	IL-1β, IL-6, IL-10, TNF-α, IFN-γ	0–12 months	Gambia	Cross-sectional	Burl et al. (191)
Whole blood	TLR2 and NOD1/2, TLR2/1, TLR3, TLR4, TLR7/8, TLR9	IL-1β, IL-6, IL-8, IL-10, IL-12p40, IL-12p70, TNF-α, IFN-α2, IP-10, IL-23, MCP-1, MIP-1α, MIP-1β, IFN-γ	0–12 months, adults 24–47 years	South Africa	Longitudinal and adults	Reikie et al. (182)
Whole blood	TLR2, TLR3, TLR4, TLR7/8 NOD1, NOD2, NALP3	IL-1β, IL-6, IL-10, IL-12, TNF-α, IFN-γ IL-8 (CXCL8), MCP-1, MIP-1α, MIP-1β, IP-10 and eotaxin (for Alum experiments)	1–18 months	Papua New Guinea	Cross-sectional	Lisciandro et al. (189)
PBMCs (fresh)	TLR1/2, TLR3, TLR4	IL-6, TNF-α	0–24 months, adults 25–40 years	Taiwan	Longitudinal (partially) and adults	Liao et al. (192)

In particular, a study on Belgian infants up to 1 year reported that the production of two type I IFN inducible chemokines, IP-10. and MIG, increases upon TLR9 stimulation in an age-dependent manner, but still remains significantly lower compared to adults (144). In line with the above, Vosters et al. (183) also found a significant increase of TLR9 induced IFN-α in early infancy, but still its levels remained below adult values up to 18 months of age. Similarly, in a longitudinal study from birth to 2 years, conducted in a North American population, TLR7/8 and TLR9 driven IFN-α2 responses increased to reach adult levels by 1 year of life, whereas the response to TLR3 stimulation decreased until the second year, to rise at a later time point to adult levels (176). A more rapid postnatal increase of IFN-α levels, that reached those of adults within the first month of life upon TLR3, 7, and 9 stimulation, was observed by Belderbos et al. (184) in a prospective study in The Netherlands. Recently, RSV-induced IFN-α production in PBMCs from human neonates and young children aged 12–59 months was found significantly lower than in adults. As expected, pDCs were identified as the main cellular source of IFN-α and, interestingly, IFN production was mediated by retinoic acid-inducible gene I protein (RIG-I) activation, thus indicating that in addition to TLR-, other PRR-mediated responses are also attenuated in early life (185). In contrast to all the above findings, a prospective study of infants from South Africa revealed a decrease of TLR7/8 and 9 mediated IFN- α2 production from a high in the first 6 months of life to an adult low by 12 months of age (182).

The innate production of IL-12, a key Th1 trophic cytokine, during early life has been assessed by many researchers and is characterized by a slow increase throughout childhood. Härtel et al. showed that the LPS-induced expression of IL-12 in monocytes increases with age (186). In a cross-sectional study from Australia, the capacity of PBMCs to synthesize IL-12p70 upon stimulation with TLR4 or heat-killed *Staphylococcus aureus*, was found impaired both in 5- and 12-year-old children, in comparison to adults (187). The same group showed that IL-12 p35 synthesis in response to TLR4 ligation matures to adult levels at some point between 4 and 13 years (188). The findings of Corbett et al. (176) are in line with the above, as in their study with North American children IL-12p70 production increased but remained below adult levels even at 2 years of age, upon TLR3, TLR4, and TLR7/8 stimulation. Trends of increasing IL-12 with age, between 1 and 18 months were also found after TLR3 and TLR4/IFNγ primed stimulation in a Papua New Guinean population (189). A more rapid maturation of IL-12 synthesis to adult levels was observed under different experimental conditions by Nguyen et al., as in their study adult levels of IL-12p70 were measured in LPS-stimulated whole blood samples by the age of 6 months (144). Belderbos et al. also showed a rapid increase of TLR3, TLR7, and TLR9 (but no TLR4) induced IL-12p70 production to adult levels, within the first month of life (184). Again, the findings from the South African cohort show an opposite trend, as TLR3 induced production of IL-12p70 in infants <6 months of age was found at levels above those of 1-year-old subjects and adults, the last two being similar (182).

Data on pro-inflammatory TNF-α and IL-1β production are also TLR-dependent and present a degree of heterogeneity. Most researchers found that their synthesis upon TLR4 stimulation is impaired in the beginning of life and increases to adult levels at some point in infancy or childhood. Belderbos et al. reported that TLR4-mediated production of TNF-α mRNA was deficient at age 1 month (184), and adult protein levels were reached at 6 months of age in Belgian children (144) and at 12 months in a Finnish cohort (190). LPS-induced TNF-α production was found still impaired in 1-year-old Australian children, but interestingly, in that study, cord blood levels were similar to those of adults with lowest levels observed at 2 months (188). In North American children both TNF-α and IL-1β increased to adult levels over the first year of life and were maintained rather stable until at least 2 years of age (176). In Gambian infants TNF-α production upon TLR4 ligation increased rapidly, within the first postnatal month, and its levels remained stable for the rest of infancy, whereas IL-1β production was high at birth and remained stable throughout infancy (191). In South African population, Reikie et al. observed a postnatal increase of both TNF-α and IL-1β levels to a peak around 12 months of age and a subsequent fall to adult levels (182). A postnatal increase of LPS-induced IL-1β was also observed in Papua New Guinea infants up to 18 months (189). Of note, this is the only study reporting an age-related decrease in LPS-induced TNF-α throughout the first year of life. On the contrary, most studies assessing pro-inflammatory responses induced by virus-mimicking TLR ligands have observed an opposite developmental pattern. Indeed, studies from different centers have shown a decline in the production of TNF-α and/or IL-1β in response to TLR7/8 and 9 stimulation from 1st to 12th month of age (144, 176, 182, 191). Poly(I:C) in these studies failed to elicit robust TNF-α and IL-1β responses and only Lisciandro et al. report an increase in TLR3 induced IL-1β levels during infancy in Papua New Guinea (189). Among other pathways assessed, it is of interest that in North American children both TNF-α and IL-1β increased from birth to 1 year of age, upon TLR2/1 stimulation (176), while in South African infants their levels decreased, as was also observed upon NOD1/2 and TLR2 stimulation (182). Finally, Lisciandro et al. have assessed innate pro-inflammatory responses to the vaccine adjuvant aluminum in Papua New Guinean infants, and reported decreasing IL-1β levels during the first 18 months of life (189).

IL-6 is a multifunctional pro-inflammatory cytokine, also implicated in supporting Th2/Th17 responses. In different studies, IL-6 cord blood levels have been found higher than those of adults, with few exceptions. Many researchers observed a drop during infancy to an adult-level nadir; this time point differed among populations and TLR stimulation. Some found that the decrease of IL-6 production to adult levels occurred around 3 months (upon TLR4 and 9 stimulation) (144) and others around 1 year (upon TLR1/2, 3 and 4 stimulation or NOD1/2 and TLR2, TLR2/1 and TLR7/8) (182, 192), 2 years of age (upon TLR2/1,4,7/8 stimulation) (176), or later during childhood (upon TLR4 and 6 stimulation) (193). Yerkovich et al. found that the first year nadir of TLR 4-induced IL-6 was followed by subsequent increase during preschool age to adult levels (188). On the contrary, some researchers found no difference between IL-6 levels between birth and 12th month of life (191) or adulthood (184), while only one group found a progressive increase in TLR2 and 3 induced IL-6 levels during infancy, in Papua New Guinean children (189).

Th17 supporting IL-23 levels have been assessed by several researchers. In North American children upon TLR4 and 7/8 stimulation, IL-23 decreased from a robust production in the beginning of life to an adult low around the 12th month of life (176). In South African children, changes were stimulus-specific with postnatal decrease of IL-23 upon TLR3 and TLR7/8 stimulation, but postnatal increase during infancy and subsequent decrease at a later time point upon TLR4 ligation (182). One group found that the TLR4 induced p19 subunit of IL-23 gradually increased, to reach adult levels between 4 and 13 years (188).

Finally, the production of anti-inflammatory IL-10, has been extensively studied. Several researchers found increased IL-10 production upon TLR4 stimulation at birth compared to adults and this robust production persisted throughout the first month (184) or the first year (144, 191) of life. Interestingly, the same pattern of enhanced production of IL-10 in the beginning of life has been also observed upon stimulation with TLR1/2, 2/1, 3, 5, 6, 7/8, 8, 9, and NOD1/2 agonists among exceedingly different populations (144, 176, 182, 184, 191, 193). Studies comprising broader age spans or older children have shown that significant falls in IL-10 levels to or below adult values occur at some later time point during infancy or childhood, with broad variation among studies, from the second month (188) to the second year (176). Subsequently, during childhood years, IL-10 production is stabilized or increased to reach adult levels. Nonetheless, there are studies from Europe (183, 190) or Papua New Guinea (189) that have shown an impairment of IL-10 production at birth and progressive age-related augmentation upon TLR4 and TLR3 stimulation, respectively.

## Future Directions

**Figure 1:**
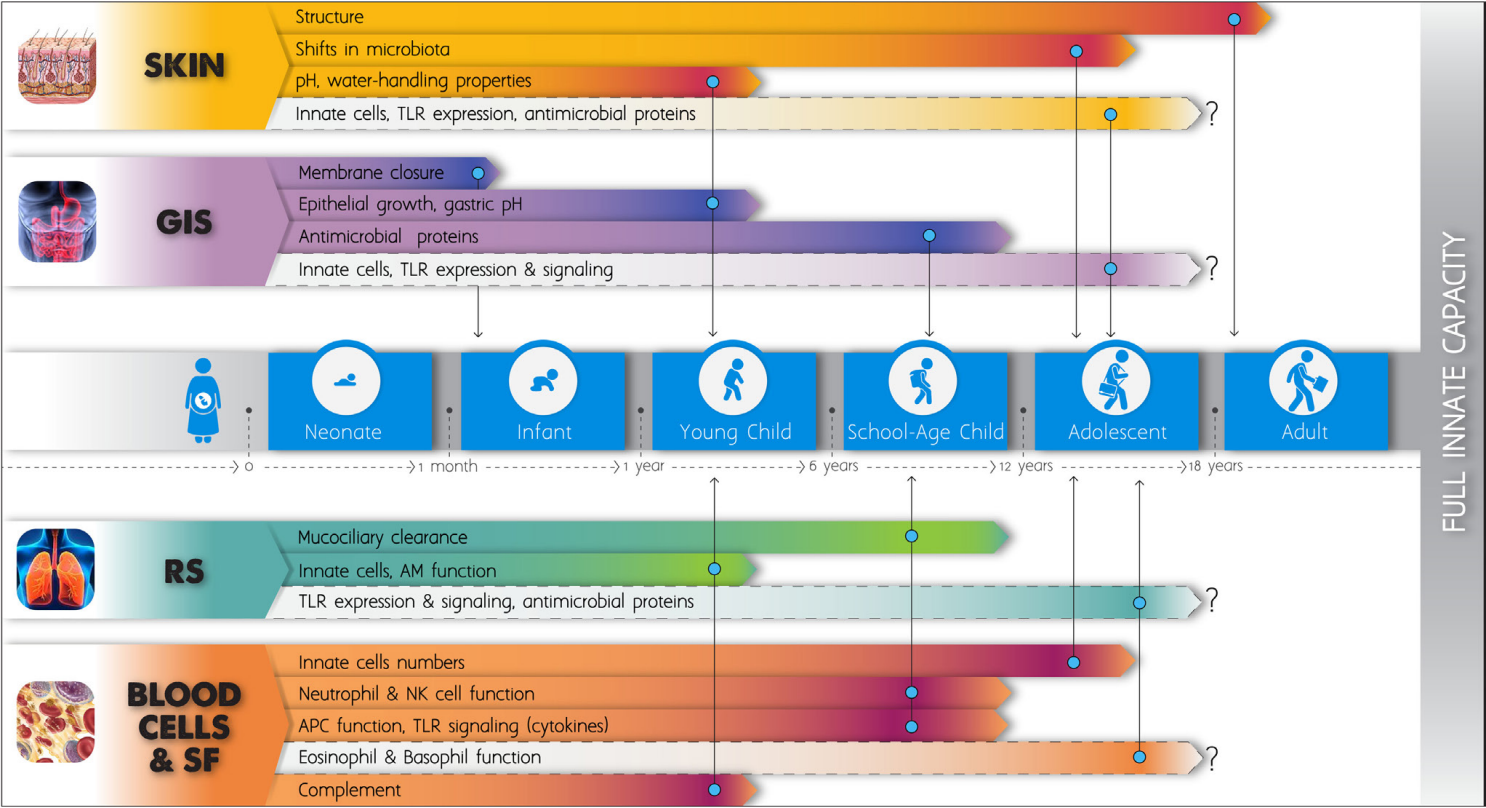
Schematic figure of the postnatal development of the innate immune system. The length of the elongated arrowheads indicates the time point at which full functional capacity is obtained and no further changes apparently occur in basic innate immune components, according to the current literature. Light color is applied to fields that remain insufficiently studied up to now. GIS, gastrointestinal system; RS, respiratory system; SF, soluble factors; TLRs, toll-like receptors; AM, alveolar macrophage; NK, natural killer; APCs, antigen-presenting cells.

Numerous significant deficiencies of the innate defense mechanisms in neonatal life have been known since the last 15 years. More recent research has demonstrated that major developmental changes occur during infancy and childhood affecting every single layer of the innate immune system, from external barriers to single cell function. Figure 1 schematically depicts key elements according to the current literature. Estimates of the age at which various functions are stabilized to adult levels vary significantly among studies, and this may be attributed to the differing methodologies, but also to the distinct characteristics of each population. Our expanding knowledge has improved our understanding on the increased susceptibility to infections in early life and is providing new opportunities for the development of novel diagnostic tools and pharmaceutical molecules. Nonetheless, much remains to be learned about age-dependent maturation of innate components in different populations. Longitudinal age-related changes in the numbers and function of innate cells in the human skin, gut, and respiratory mucosae have not been sufficiently assessed. No data on functional maturation of basophils and eosinophils exist. Developmental differences in the expression of antimicrobial molecules in mucosal surfaces require further study. The recent findings on the postnatal ontogeny of TLR-mediated cytokine responses in human blood need to be expanded, as most studies have up to now focused only on changes occurring during the first months of life. Studies engaging children older than 2 years have mainly assessed the maturation of the TLR4 pathway (186–188, 194) and only two assessed additional TLR pathways up to the age of five (181, 193).

Furthermore, our understanding of how genetic and environmental factors determine immune trajectories has only recently began to emerge (195). In a longitudinal study, Garand et al. assessed responses following TLR stimulation in infants across two distinct racial groups, Asian and Caucasian, growing up in the same environment. They found that differences present at birth between the two groups disappeared by the first or second year, and this finding highlights the importance of environmental influences upon the innate developmental process (196). Netea et al. proposed that repeated stimuli may result in an enhanced innate function (termed “trained immunity”), mediated by epigenetic mechanisms (197). Prenatal and early-life exposures might shape/reprogram the immune function and there is evidence arising mainly from epidemiologic studies in humans showing that these exposures may be implicated in the pathogenesis of immune-related diseases (198). Several environmental factors, such as nutrients, chemicals, and infectious agents (195, 199), have been linked to changes in innate immune function but only a few have been simultaneously associated with the pathogenesis of immune-mediated diseases. In this direction, the pathogenesis of allergic diseases has been a field of active research. The impact of antenatal exposure to farm environments has been investigated by the PASTURE and PARSIFAL studies. Roduit et al. showed that maternal contact with farm animals and cats during pregnancy has a protective effect on the offspring in relation to the development of atopic dermatitis, associated with a lower expression of innate immune receptors at birth (200). In the children of PARSIFAL, both atopic sensitization and the gene expression of receptors of innate immunity were strongly determined by maternal exposure to stables during pregnancy (201). Furthermore, post-utero farm living and other specific early-life exposures, such as breastfeeding and day-care attendance have been associated with reduced subsequent risk of allergy through innate immune mechanisms, at least to a certain extent (10, 202). Although much is to be learnt on how these mechanisms exert their beneficial effect, the role of DCs seems to be pivotal. Recent data suggest that farm environment may modify the relative proportions of DC subsets during childhood (by specifically reducing mDC1or mDC2 percentages) and show that functional properties of mDCs are positively associated with asthma (203, 204). Other studies have pointed out differences in innate immune function between allergic and non-allergic children at various ages (205, 206), and Prescott et al. observed increased pro-inflammatory TLR responses in newborns who subsequently developed food allergy and atopic dermatitis (207). The alteration of the antiviral responses in the atopic background has been investigated during the last decade (208–210) and still remains a field of active research, especially in relation to respiratory allergic diseases (211–215). Innate immunity is implicated in the pathogenesis of atopic eczema (216), asthma (217, 218), and allergic rhinitis (219) and recent research focuses on gene–environment interactions (220, 221). Much is to be learned from longitudinal studies. Tulic et al. observed that the occurrence of allergic diseases correlates with differential developmental innate responses (181). As recent data suggest the implication of innate immunity in the pathogenesis of immune-mediated diseases other than allergy, such as type 1 diabetes (222, 223), celiac disease (224), inflammatory bowel disease (225), rheumatoid arthritis (226), and psoriasis (227), it is of great importance to gain insight into normal immune development and into the role of environmental influences. In the years to come, in depth characterization of the innate immune maturation and interfering factors may help us discover windows of vulnerability and provide new evidence on disease pathogenesis but also for possible interventions to reduce morbidity.

## Author Contributions

AG contributed to the conception and design of the review, the acquisition of data, the drafting of the article, and the approval of the version to be published. NP contributed to the conception and design of the review, the drafting of the article, its revision, and the approval of the version to be published before submission. Both authors agree to be accountable for the content of the work.

## Conflict of Interest Statement

NP has received grants from Nestle and Menarini for research support, consultancy fees from Novartis, Menarini, MEDA, ALK-Abello, GSK, and Chiesi, honoraria from FaesFarma, Novartis, Uriach, Abbvie, Stallergenes, MSD, MEDA, Omega Pharma Hellas, and Nutricia and is in the Scientific Advisory Board of Novartis, FaesFarma, BioMay, HAL, Nutricia, MEDA. AG declares that the research was conducted in the absence of any commercial or financial relationships that could be construed as a potential conflict of interest.
